# Gastric Candidiasis Leading to Perforation: An Unusual Presentation

**DOI:** 10.7759/cureus.17878

**Published:** 2021-09-10

**Authors:** Mallesh Kavyashree, Bishal Pal, Souradeep Dutta, Bhawana Ashok Badhe, Vishnu Prasad Nelamangala Ramakrishnaiah

**Affiliations:** 1 Surgery, Jawaharlal Institute of Postgraduate Medical Education and Research, Puducherry, IND; 2 Pathology, Jawaharlal Institute of Postgraduate Medical Education and Research, Puducherry, IND

**Keywords:** pneumoperitoneum, candida tropicalis, stomach, fungal perforation, gastric perforation

## Abstract

Candidal infection of the gastrointestinal tract (GIT) is rare but has recently increased due to the increased number of immunocompromised patients, injudicious use of antibacterial agents, and prolonged use of antacid drugs in immunocompetent patients. The most frequent organ involved in GIT candidiasis is the esophagus, followed by the stomach, small intestine, and large intestine. The clinical spectrum of gastric candidiasis ranges from asymptomatic to gastric perforation and even shock. This case report presents a 58-year-old immunocompetent male patient diagnosed with *Candida tropicalis-*induced gastric perforation peritonitis.

## Introduction

Fungal organisms are commensals of the gastrointestinal tract (GIT). *Candida* species carriage in healthy individuals ranges from 30% to 60% [1]. Species like *Candida albicans*, *Candida tropicalis*, *Candida glabrata*, *Candida parapsilosis*, and *Candida krusei* are common conditionally pathogenic fungi that cause gastrointestinal infection in immunocompromised and debilitated patients [2]. *Candida tropicalis* is widespread in the environment, human skin, and digestive tract, and rarely causes localized and systemic infection [3].

Gastrointestinal candidiasis most commonly involves the esophagus followed by the stomach, small intestine, and large intestine. Nearly 12% of all cases of peritonitis are fungal peritonitis, and *Candida* is the most frequent agent identified [4]. It occurs mainly in immunosuppressed conditions like hematological or other malignancies on chemotherapy, transplant patients and patients on long-term steroid therapy, diabetes mellitus, and HIV infection, and is rare in healthy persons [5,6]. Significant risks for the development of Candidal peritonitis include hollow viscus perforation, abdominal and thoracic surgery, drain in situ, antibiotic therapy more than 48 hours before peritonitis, and extensive *Candida* colonization. Here, we present a case of gastric prepyloric perforation due to *Candida tropicalis *in an immunocompetent patient.

## Case presentation

A 58-year-old hypertensive gentleman from Cuddalore, Tamil Nadu, presented with acute onset, severe, progressive epigastric pain for two days. He complained of two to three episodes of bilious vomiting, abdominal distension, and obstipation for two days. He also had complaints of reduced urine output for two days. There was no history of fever, jaundice, hematemesis, melena, or previous surgeries. He was an occasional alcoholic for the past 30 years. Two months back, he was evaluated in a different hospital with complaints of recurrent abdominal pain. He underwent upper gastrointestinal endoscopy there, which showed a large duodenal ulcer with deformed duodenum. Endoscopic biopsy showed features of chronic duodenitis, for which he was prescribed long-term proton-pump inhibitors.

**Figure 1:**
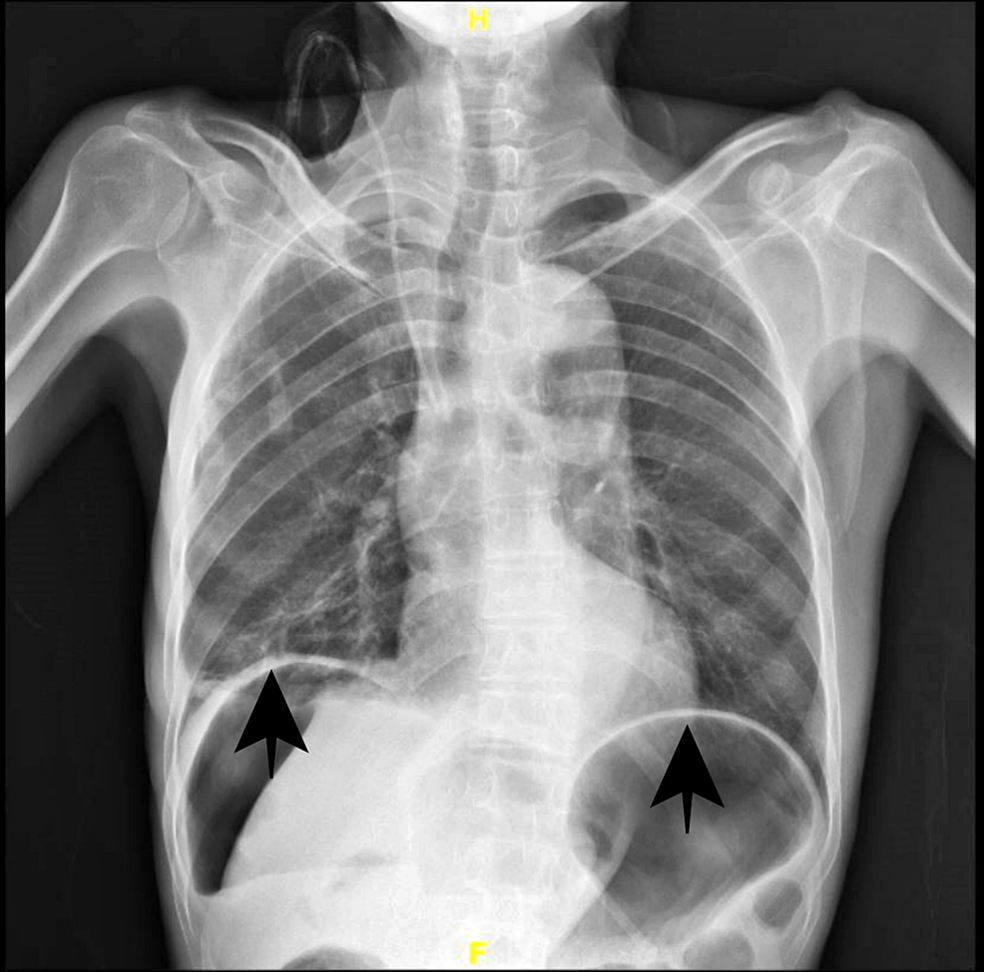
Chest X-ray showing air under the diaphragm (black arrows).

He was dehydrated and tachycardic (pulse rate 112/min) but maintained blood pressure (150/110mmhg). His abdomen was rigid with generalized tenderness and rebound tenderness; bowel sounds were sluggish on auscultation. His blood parameters showed haemoglobin - 12.4 gm/dl, total leucocyte count - 9720/cubic mm with neutrophils of 81%, urea - 167 mg/dl, creatinine - 3.42 mg/dl, sodium - 127 mmol/L, and potassium - 4.2 mmol/L. Chest X-ray showed air under the diaphragm (Figure 1).

**Figure 2:**
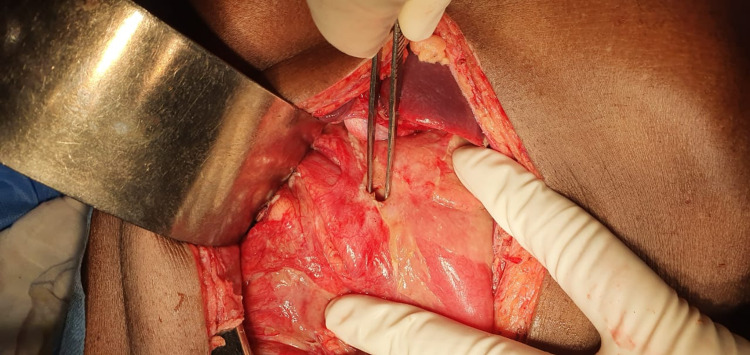
Intraoperative picture showing 5 x 5 mm perforation in the prepyloric region of the stomach.

With a diagnosis of peptic perforation, he was wheeled in the operating room for an urgent laparotomy. Intraoperatively a perforation of size 5 mm x 5 mm was found in the pre pyloric region of the stomach with multiple pus flakes present over the surface of the liver and stomach with flimsy adhesions present between them (Figure 2).

**Figure 3:**
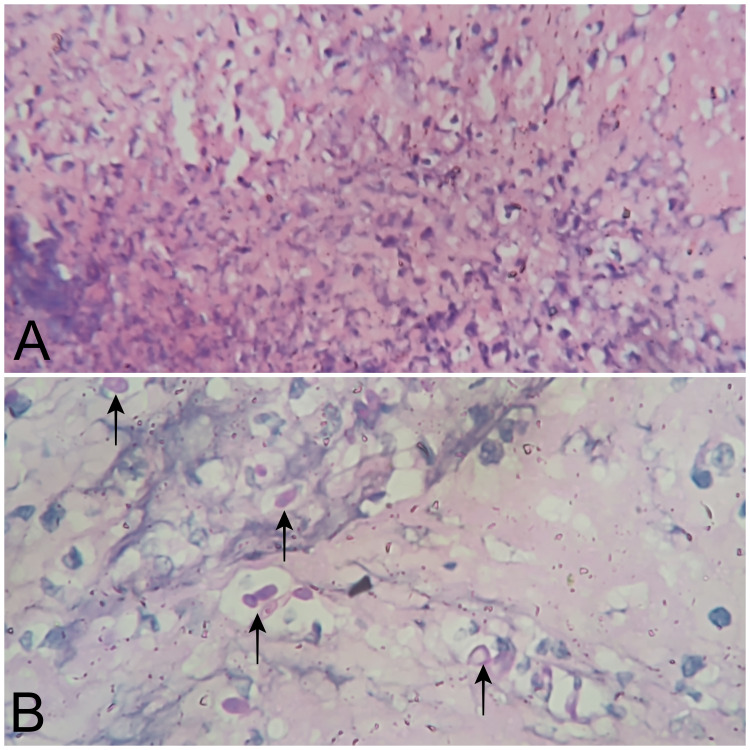
(A) Microscopic section of the stomach wall showing acute inflammatory granulation tissues (H&E x200). (B) Microscopic section of the stomach wall showing acute inflammatory cells along with PAS-positive fungal organisms in yeast and pseudohyphal forms (PAS x400). H&E: haematoxylin and eosin; PAS: periodic acidic-Schiff. Black arrows showing PAS-stained yeast and pseudohyphal forms.

An edge wedge biopsy was taken from the ulcer edge. Modified Graham's patch repair was done. He received seven days course of empirical antibiotics (cefoperazone-sulbactam with metronidazole). He was empirically started on fluconazole as his intraoperative abdominal fluid grew *Candida tropicalis*. Wedge biopsy revealed acute inflammatory granulation tissue with the adjacent congested gastric mucosa. Periodic acid-Schiff (PAS) staining highlighted yeast and pseudohyphal forms of *Candida* (Figure 3).

Later antibiotic sensitivity test identified *Candida tropicalis* sensitive to fluconazole, and fluconazole was continued for 14 days. He improved symptomatically, and the rest of the hospital stay was uneventful.

## Discussion

*Candida* species are ubiquitous fungi and are well niched in the gastrointestinal habitat of a healthy human. Their presence is generally benign. *Candida* species cause infection mostly in immunocompromised and debilitating patients like diabetes, patients on long-term steroid therapy and patients on chemotherapeutic drugs, and HIV/AIDS patients [7], and rarely in healthy individuals with high-risk factors like prolonged antacid use [8]. Any predisposing factor can cause a loss in the mucosal barrier and subsequent low level of inflammation in GIT, which increases *Candida* colonization and promotes inflammation again. This vicious cycle results in delayed healing of ulcers and may even invade tissues that can progress to perforation [9]. Usually, this fungal colonization is controlled by the beneficial bacterial flora and low pH of GIT. Improper use of antibacterial agents causing an imbalance in the bacterial-fungal colony, prolonged use of antacids resulting in an increase in gastric pH, and hyperglycemia facilitating fungal colonization predispose an immune-competent host for fungal infection, most frequently *Candida* infection [8]. Other risk factors that influence this colonization to infection are outlined in Table 1.

**Table 1:** Risk factors that influence Candida colonization to infection.

Risk factors that influence *Candida* colonization to infection	
*Candida*-specific factors	Stain and antifungal susceptibility
	Size and site of infection
Host-specific factors	Co-morbidities reducing host immunity like diabetes and HIV
	Malnutrition and resulting mucosal breach
Iatrogenic factors	Widespread antibiotic use
	Instrumentation of gastrointestinal tract
	Surgical intervention resulting in mucosal injury

Peptic ulcer disease (PUD) is one of the most commonly encountered diseases. Frequent causes of PUD are intake of pain medications like nonsteroidal anti-inflammatory drugs, *Helicobacter pylori* infection, alcohol and tobacco abuse, severe stress, bacterial and viral infection, head injury, shock, burns, radiation therapy, Zollinger-Ellison syndrome, and infrequently fungal infection [7]. Although generally rare, the prevalence of *Candida* infection of the gastrointestinal tract has significantly increased in recent years due to increased immunocompromised individuals and injudicious use of antibacterial agents. Two large-scale studies done by Eras et al. and Tsukamoto et al. mentioned that incidence of GIT mycosis was 4.35% and 5.9% subsequently, and the most frequent organ involved was the esophagus, followed by the stomach, the small intestine, and the large intestine [10]. Minoli et al., in their study of gastric candidiasis, found that around 0.96% of all patients undergoing endoscopy were affected by candidiasis and most of these patients were elderly males [11]. Jindal et al., in their study of the culture of abdominal fluid of 140 patients presented with gastrointestinal perforation, found that 68 specimens (i.e., 48.6%) were positive for *Candida*. *Candida albicans* presents in 52 out of 68 specimens (i.e., 76.5%) and non-albicans *Candida* presents in 16 out of 68 specimens (i.e., 23.5%) with *C. krusei* in 8 (6.5%) specimens, *C. tropicalis* in 4 (3.2%) specimens, and *C. glabrata* in 3 (2.4%) specimens [12].

Gastric candidiasis may be of thrush, nodular, ulcerative form. Thrush type presents a readily removable white or greenish-white membrane that spreads over inflamed mucosa and usually involves other areas like oral thrush. The nodular form presents nodular projections of few millimeters over highly inflamed mucosa, usually in the antral region. The ulcerative form presents like other gastric ulcers with no specific endoscopic findings and is most commonly reported in the literature. Still, there are arguments whether, in ulcerative form, *Candida* infection is the cause of the ulceration or ulcer gets infected secondarily during its progression. Zwolińska-Wcisło et al. in their study concluded that fungal colonization is secondary [13]. Most gastric candidiasis patients present with ulcer-like pain, nausea, vomiting, and weight loss, and in extreme cases, like ulcerative forms, gastrointestinal bleeding, perforation peritonitis, and even shock with multiorgan failure. *Candida* peritonitis can be community-acquired or nosocomial. Nosocomial infections are defined as *Candida* peritonitis more than 48 hours after admission and are more severe with more resistant *Candida* agents as causative. There are no specific investigations other than upper gastrointestinal endoscopy and endoscopic biopsy or growth of *Candida* in culture media to diagnose gastric candidiasis. Non-specific laboratory findings include neutrophilic leucocytosis, high C-reactive protein, and high procalcitonin.

A conclusive diagnosis of gastric candidiasis can be made based only on histopathological findings of fungal hyphae with microbiologically confirmed *Candida* species. Histological staining using either the PAS or Gomori's methenamine silver stains demonstrate Candidal elements like septate hyphae with characteristic dichotomous branching (at an angle of approximately 45°) and yeast cells. Non-specific inflammation of the surrounding area is a common histopathological finding in all forms. Additional findings like thrush types present with no or diffuse mycotic infiltration, nodular types have mycotic granuloma formation, ulcerative types present as mycotic micro abscess, and mycotic granuloma with widespread mycotic infiltration [11]. Isolation of *Candida* in body fluid or tissue samples in peri-operative samples is more clinically significant than postoperative samples. Serological tests like detection of antibodies against (1,3)-beta-D-glucan, galactomannan, and mannan can be used with variable sensitivity and specificity. Polymerase chain reaction (PCR), a DNA genomic amplification test, detects *Candida* antigen with high sensitivity and specificity. A specialized semi-nested PCR (snPCR) has been developed to improve the sensitivity of traditional PCR by 10 times with a detection limit of less than 10 yeast cells per sample. MALDI-TOF (matrix-assisted laser desorption ionization-time of flight) is a mass spectrometry assay, which is a new diagnostic test that can identify *Candida* strains in approximately one hour, but with limitations [14].

The current guidelines do not recommend empirical antifungal use for all gastric candidiasis patients except patients of recurrent perforations, recent abdominal surgery, anastomotic leaks, and critically ill patients requiring ICU care and ventilator patients [15]. In-hospital stay of more than 29 days is a strong predictor for fluconazole-resistant *Candida* isolation among critically ill emergency surgery patients [16]. For *Candida* albicans, fluconazole with a dose of 400 mg per day is an appropriate choice, but in fluconazole-resistant *Candida* species and critically ill patients, echinocandin-like caspofungin with a dose of 50 mg per day is recommended. Amphotericin B is not recommended as initial therapy due to its toxicity [17]. A minimum of two weeks of antifungal agents is recommended after documented clearance of fungal agents in peritoneal fluid or blood samples. Therapeutic failure is considered if there is no improvement clinically, persistent positive fungal culture, and persistent raised inflammatory markers of inflammation such as neutrophilia and raised C-reactive protein even after three to four days of starting antifungal agents. Second-line agents, combined therapy, or surgical management must be carried out in case of therapeutic failure. Sometimes *Candida* colonization scores like "*Candida* score" may be used to identify who should receive antifungal prophylaxis in critically ill patients like patients on parenteral nutrition, recent abdominal surgery, and severe sepsis patients [18].

Surgery is mandated when a patient presents with a visceral perforation following perforation a fungal ulcer. Identification of visceral abscess and surgically draining is necessary for the treatment of peritonitis. Otherwise, conservative managements like removing all possible sources like intravascular and urinary catheters, intraabdominal drains, and prosthetic materials have to be removed because fungi can form biofilms over it [4]. Our patient presented with perforation peritonitis, for which he underwent surgery and had *Candida tropicalis* in both gastric ulcer and peritoneal fluid. We started fluconazole empirically, which was later reported as sensitive to fluconazole, and a full 14 days course was completed.

## Conclusions

Gastric candidiasis is a sporadic infection that can turn severe and can cause perforation in both immunocompromised as well as immunocompetent patients if diagnosed late. High clinical suspicion and early diagnosis are fundamental in the management. We hope to increase awareness among physicians and surgeons to include candidiasis as a differential diagnosis in a patient with gastrointestinal perforation.
